# Magnetoencephalography Detection of High-Frequency Oscillations in the Developing Brain

**DOI:** 10.3389/fnhum.2014.00969

**Published:** 2014-12-12

**Authors:** Kimberly Leiken, Jing Xiang, Fawen Zhang, Jingping Shi, Lu Tang, Hongxing Liu, Xiaoshan Wang

**Affiliations:** ^1^Department of Pediatrics, Magnetoencephalography (MEG) Center, Cincinnati Children’s Hospital Medical Center, Cincinnati, OH, USA; ^2^Department of Neurology, Cincinnati Children’s Hospital Medical Center, Cincinnati, OH, USA; ^3^Department of Communication Sciences and Disorders, University of Cincinnati, Cincinnati, OH, USA; ^4^Department of Neurology, Nanjing Brain Hospital, Nanjing Medical University, Jiangsu, China

**Keywords:** magnetoencephalography, high-frequency oscillations, somatosensory cortex, wavelet, beamformer, pediatrics

## Abstract

Increasing evidence from invasive intracranial recordings suggests that the matured brain generates both physiological and pathological high-frequency signals. The present study was designed to detect high-frequency brain signals in the developing brain using newly developed magnetoencephalography (MEG) methods. Twenty healthy children were studied with a high-sampling rate MEG system. Functional high-frequency brain signals were evoked by electrical stimulation applied to the index fingers. To determine if the high-frequency neuromagnetic signals are true brain responses in high-frequency range, we analyzed the MEG data using the conventional averaging as well as newly developed time-frequency analysis along with beamforming. The data of healthy children showed that very high-frequency brain signals (>1000 Hz) in the somatosensory cortex in the developing brain could be detected and localized using MEG. The amplitude of very high-frequency brain signals was significantly weaker than that of the low-frequency brain signals. Very high-frequency brain signals showed a much earlier latency than those of a low-frequency. Magnetic source imaging (MSI) revealed that a portion of the high-frequency signals was from the somatosensory cortex, another portion of the high-frequency signals was probably from the thalamus. Our results provide evidence that the developing brain generates high-frequency signals that can be detected with the non-invasive technique of MEG. MEG detection of high-frequency brain signals may open a new window for the study of developing brain function.

## Introduction

Increasing evidence indicates that the brain generates both very low- and very high-frequency brain signals (Bowyer et al., 2012; Zijlmans et al., 2012; Haegelen et al., 2013). Low-frequency brain signals are typically referred to as “direct current” or “infraslow activity” (Bowyer et al., 2012). High-frequency brain signals are also called “high-gammas” (Huo et al., 2010), “high-frequency oscillations” (HFOs), “ripples,” or “fast ripples” (Worrell et al., 2008; Engel et al., 2009; Gotman, 2010; Haegelen et al., 2013). High-frequency brain signals are potential new biomarkers for the study of brain function (Ozaki and Hashimoto, 2011).

In comparison with conventional low-frequency brain signals (<70 Hz), high-frequency brain signals are typically very weak (low in amplitude) (Worrell and Gotman, 2011). Neural signals from the brain are the spatiotemporal summation of synchronous firing from at least 10,000–50,000 neurons (Murakami and Okada, 2006). Therefore, it has been postulated that the functional organization of brain activity is encoded in multi-frequency ranges of that neuronal activity (Lina et al., 2014). The detection and recording of such high-frequency brain signals requires a high-sampling rate. Therefore, how to handle the large datasets digitized at a high-sampling rate has become an important issue for those investigating these signals (Worrell et al., 2012). In particular, using non-invasive techniques to detect and localize very high-frequency brain signals is rapidly becoming a new technique in many areas of research and clinical work (Miao et al., 2014; Rampp et al., 2014).

The most recognized HFOs are identified in the somatosensory cortex (Kotecha et al., 2009b). Previous reports have shown that the early portion of this activity is generated by action potentials of thalamocortical fibers and the late somatosensory HFO burst results from these action potentials arriving at the somatosensory cortices; specifically Brodmann areas 3b and 1 (Ozaki and Hashimoto, 2005). Though the exact range of HFOs remains unclear, HFOs up to 2632 Hz are detectable from the human somatosensory cortex with invasive recordings (Sakura et al., 2009). Since these oscillations are very weak, previous studies have required more than 1000 trials to obtain reliable results from adults (Ozaki et al., 1998). However, modern magnetoencephalography (MEG) systems have a whole-cortex sensor array, which can capture the spatial information of brain activity from variety of angles (Barkley and Baumgartner, 2003). This mechanism allows HFOs to be detected and localized with a much smaller number of trials. Time-frequency analysis and spatial filtering (Kotecha et al., 2009b), described in further detail below, can be used for HFO investigations with MEG.

The objective of the present study was to assess high-frequency brain signals in children using newly developed MEG methods. The present paper will detail the mathematic algorithms of our new MEG analysis technique. Our central hypothesis was that somatosensory HFOs can be non-invasively localized using wavelet-based source localization. In comparison to previous reports on high-frequency brain signals (Jacobs et al., 2012), the major innovation of the present study was the optimization of MEG approaches for localizing very high-frequency neuromagnetic signals (>1000 Hz) in the developing brain. We consider this study of paramount importance because the development of reliable high-frequency signal detectors will likely have a significant impact on future clinical applications of MEG, such as presurgical brain mapping in those suffering from epilepsy (Gloss et al., 2014).

## Materials and Methods

### Subjects

Twenty healthy children (age range: 6–17 years, mean and SD: 12.3 ± 2.7 years, 10 girls and 10 boys) were recruited for this study. Inclusion criteria were (1) healthy without a history of neurological disorders or brain injuries; (2) age-appropriate levels of hearing, vision, and hand movement. Exclusion criteria were (1) inability to remain still inside the MEG scanner; and (2) presence of any non-removable metal device, such as a cochlear implant, a pacemaker, or a neurostimulator containing electrical circuitry, generating magnetic signals, or having other metal that could produce visible magnetic noise in the MEG data; (3) visually identifiable magnetic noise in the subject’s recording (amplitude of waveforms >6 Pt). Since head movement during MEG recording could affect the accuracy of source estimation, data from a subject with head movement larger than 5 mm would be excluded from analysis. Written consents, formally approved by the Institutional Review Board (IRB) at Cincinnati Children’s Hospital Medical Center (CCHMC) or Nanjing Brain Hospital, were obtained from each participant prior to testing.

### Stimulus

Two Digitimer Constant Current Stimulator model DS7A electrical stimulation systems (Digitimer Ltd., Welwyn Garden City, England) were used to stimulate the participant’s right and left index fingers independently via Digital Ring Electrodes (Oxford Instruments Medical, Hawthorne, NY, USA). One hundred stimuli were delivered to each finger. The duration of the electrical stimulus was 0.3 ms (Kotecha et al., 2009b). The interval between two stimuli was 1010–1030 ms as the electrical stimuli were randomized by varying interstimulus intervals (10 ~ 30 ms) using the software BrainX (Kotecha et al., 2009b). The stimuli were adjusted to an intensity that delivered stimulation large enough to be detected by the participant yet not cause pain, as determined by previous studies (Kotecha et al., 2009b).

### MEG recordings

Magnetoencephalography signals were recorded in a magnetically shielded room using a whole head CTF 275-Channel MEG system (VSM MedTech Systems Inc., Coquitlam, BC, Canada) in the MEG Center at CCHMC and at Nanjing Brain Hospital. Before data acquisition commenced, three electromagnetic coils were attached to the nasion, left and right pre-auricular points of each subject. These three coils were subsequently activated at different frequencies for measuring each subject’s head position relative to the MEG sensors. Each subject lay comfortably in the supine position with his or her arms resting on either side, during the entire procedure. For the study of somatosensory evoked magnetic fields (SEFs), 100 trials were recorded for each finger (200 trials for each subject). The duration of each trial recording was 1000 ms (400 ms pre-stimulation baseline and 600 ms post-stimulation time-window). The sampling rate of the MEG recording was 6000 Hz per channel. MEG data were recorded with a noise cancelation of third-order gradients and without online filtering. To identify system and environmental noise, we routinely recorded one MEG dataset without a subject immediately prior to the experiment.

### Magnetic resonance imaging scan

Three-dimensional magnetization-prepared rapid acquisition gradient echo (MP_RAGE) sequences were obtained for all subjects with a 3-T scanner (Siemens Medical Solutions, Malvern, PA, USA). Three fiducial points were placed in identical locations to the positions of the three coils used in the MEG recordings, with the aid of markers and digital photographs, to allow for an accurate co-registration of the two data sets. Subsequently, all anatomical landmarks digitized in the MEG study were made identifiable in the MR images. Pediatric brain templates developed by the MEG Center at CCHMC were also used for data comparison and visualization (Kotecha et al., 2009a).

### Waveform and time-frequency analyses

We visually inspected our MEG data and marked any possible artifacts over 6 Pt before data analyses. MEG waveforms evoked by 100 finger stimuli were averaged together over each finger for analyzing neural response amplitude and latency. Since somatosensory evoked activation has mainly been found in the range of 10–120 ms (Kotecha et al., 2009b), our data analyses focused on this latency range. To analyze high-frequency brain signals in waveforms, we performed conventional waveform filtering (Kotecha et al., 2009b) with band-pass filters of 1–20, 20–500, 500–1000, and 1000–2000 Hz. Since somatosensory evoked MEG data were digitized at a sampling rate of 6000 Hz, a band-pass filter of 2000–3000 Hz was also applied to the data. In this study, Butterworth filters were used (the phase shift was 0; the slope was −24 dB/oct).

The analysis technique of Morlet continuous wavelet transform was employed to transform time-domain data to time-frequency-domain data. The Morlet wavelet was used because brain activity is non-stationary and the wavelet is better suited for non-stationary data (Ghuman et al., 2011). The Morlet wavelet is described by the following equation:

(1) $$G\left(t,f\right)=C_{\sigma }\pi^{-\frac{1}{4}}e^{-\frac{1}{2}t^{2}}\left(e^{i\sigma t}-\kappa_{\sigma }\right)$$

In the above formula, *t* indicates time and *f* indicates frequency (or a scale in the wavelet mother function for a specific frequency). Each wavelet transform has its own sigma value. κ_σ_ represents the admissibility and *C*_σ_ represents a normalized constant. If signals appeared in the given time-sensitive (a small sigma value) and frequency-sensitive (a large sigma value) ranges, they would be enhanced.

### Forward and inverse solutions

To detect MEG signals at source levels, a three-dimensional source grid (3D grid) was developed. In the 3D grid, each grid node represented a possible source. Differing from the conventional volumetric source imaging or distributed source map, each grid node consisted of multiple data items including the strength and frequency of the source activity. Similar to previous reports (Mosher and Leahy, 1998; Vrba and Robinson, 2001; De Gooijer-Van De Groep et al., 2013), the sources of activity were determined with following equations:

(2) B=LQ+N

In Eq. 2, *B* represents the MEG data, *L* represents the lead field, *Q* represents the source strength, and *N* represents the noise. For a given MEG dataset, *B* is known and *L* can be computed for each node with a forward solution. The forward solution in this study was computed according to Sarvas’ formula for outside hemispherical conductors in Cartesian coordinates (Sarvas, 1987).

The determination of source strength and orientation of *Q* has been a challenge as discussed in many previous reports (Mosher et al., 1999; Huang et al., 2004; Robinson, 2004; De Munck and Bijma, 2009; Ou et al., 2009). According to our tests, for a given MEG dataset in multiple frequency ranges within a limited time window, the positions of the sensor array and the 3D source grid were fixed; consequently, lead fields could be computed once and then used for both low- and high-frequency ranges. Under these assumptions, we propose using single value decomposition (SVD) to decompose the lead field as in the following:

(3) $$L=\mathrm{US}V^{T}$$

Where *U* ∈*R*^mxm^ is an orthogonal (unitary in the complex case) matrix. The columns of *U* are the left singular vectors of *L*. *V* ∈*R*^mxm^ is an orthogonal matrix. The columns of *V* are right singular vectors of *L*. *S* = diag (σ_1_, σ_2_, … , σ*_p_*) is an *M* × *N* diagonal matrix with *p* = min (*m*, *n*) and σ_1_, σ_2_, … , σ*_p_* are the singular values of *L*. *M* indicates the number of sensors and *N* indicates the number of source orientations. For a single source, σ is <=3. The Moore–Penrose pseudo-inverse of *L* is given by:

(4) $$L^{+}=VS^{+}U^{T}$$

Where *S*^+^ is a diagonal formed by the multiplicative inverses of the non-zero singular values of *L*. The correlation between measured MEG data, *B*, and the lead field is defined by:

(5) $$\begin{array}{ll} B & =\mathrm{LQ}=\mathrm{US}V^{T}Q \end{array}$$

(6) $$Q=BL^{-1}$$

By replacing *L*^−1^ in Eq. 6 with *L*^+^ in Eq. 4, the estimated moment $\vec{Q}$ can be computed with a SVD back substitution:

(7) $$\vec{Q}=\mathrm{BV}S^{+}U^{T}$$

Of note, *L*^+^ could be computed once and used for the analysis of data in all frequency ranges, which makes the computation of source strength and probability more efficient. The probability of source activity was assessed with the correlation *t* value that was computed for the measured MEG signal and the computed MEG signals with Eq. 7. The threshold for correlations was statistically established to be 0.6. The equation for computing the statistical *t* is:

(8) $$t=\frac{r}{\mathrm{sqrt}\left[\left(1-r^{2}\right)∕\left(N-2\right)\right]}$$

Where *r* represents the correlation coefficients and *N* indicates the number of samples. The parameters used for establishing the threshold are (a) sample size; (b) alpha value (0.05); (c) two-tailed test; and (d) type of correlation coefficient: Pearson’s correlation. Of note, each voxel in our magnetic source imaging (MSI) had multiple values, which included source strength and source probability.

### Magnetic source imaging

To go beyond localizing magnetic sources with aforementioned algorithms, we also developed a new technique, accumulated source imaging (ASI), to localize high-frequency signals. ASI was based on the previous observation that an accumulated spectrogram of virtual sensors could reliably detect high-frequency signals (Xiang et al., 2004, 2009a,b; Xiang and Xiao, 2009). ASI was defined as the volumetric summation of source activity over a period of time. In the present study of somatosensory evoked signals, ASI was the summation of source activity of all electrical pulse trials on each finger. ASI can be described as the following equation:

(9) $$\mathrm{Asi}\left(r,s\right)=\sum_{t=1}^{n}Q\left(r,t\right)$$

In Eq. 9, ASI represents accumulated source strength at location *r*; *s* indicates the time slice; *t* indicates time point of MEG data; *n* indicates the number of trials; and *Q* indicates the source activity at source *r* and at a specific trial *t*. From a computer program point of view, the use of computer memory and storage space by Eq. 9 is dependent on the *s* for a fixed source imaging configuration (e.g., spatial resolution and dimension). Even though *n* could be infinitely increasing, the requirements for computer memory and storage remain the same. Consequently, the approach automatically avoided possible “overflow” or “out-of-space” problems in a large number of trials. The basic principle of ASI was that high-frequency MEG signals from the brain activity were locked to a spatial location and certain frequency bands. By accumulating all the source data computed for each location and each frequency band from multi-trial recordings, noise in a random space and frequency would be minimized and the signal-to-noise ratio in source imaging would then be increased.

Since this method spatially accumulates the results of source data, it is different from previously employed methods, which compute a covariance matrix or kurtosis of sensor data for a long recording. Specifically, using a covariance matrix or kurtosis for source localization is based on the assumption that the source was stationary during the long recording. Our approach, on the other hand, did not make this assumption, thereby taking into account minimal head movement typically found in pediatric MEG recordings. Therefore, our approach had the capability to detect both stationary and non-stationary source activity. Since high-frequency signals are very weak, we implemented the algorithms with C/C^++^ in double precision (64 bits). Therefore, the combination of spatial accumulation and double precision computation are well-suited to detect high-frequency signals over the course of at least 100 trials.

### Statistics

Statistical comparisons between different frequency bands were performed using a Student’s *t*-test. The normality of MEG data was tested with the Shapiro–Wilk test. For MEG data, which not found to be normally distributed, Mann–Whitney tests were used for comparisons. Statistical analyses were performed using SPSS version 16.0 for Windows (SPSS Inc., Chicago, IL, USA). The threshold of statistical significance for differences was set at *p* < 0.05. For multiple comparisons, a Bonferroni multiple comparisons correction was applied. Therefore, for the comparisons of 4 frequency bands, the *p* value would need to decrease to 0.012 (0.05/4).

## Results

**Figure 1:**
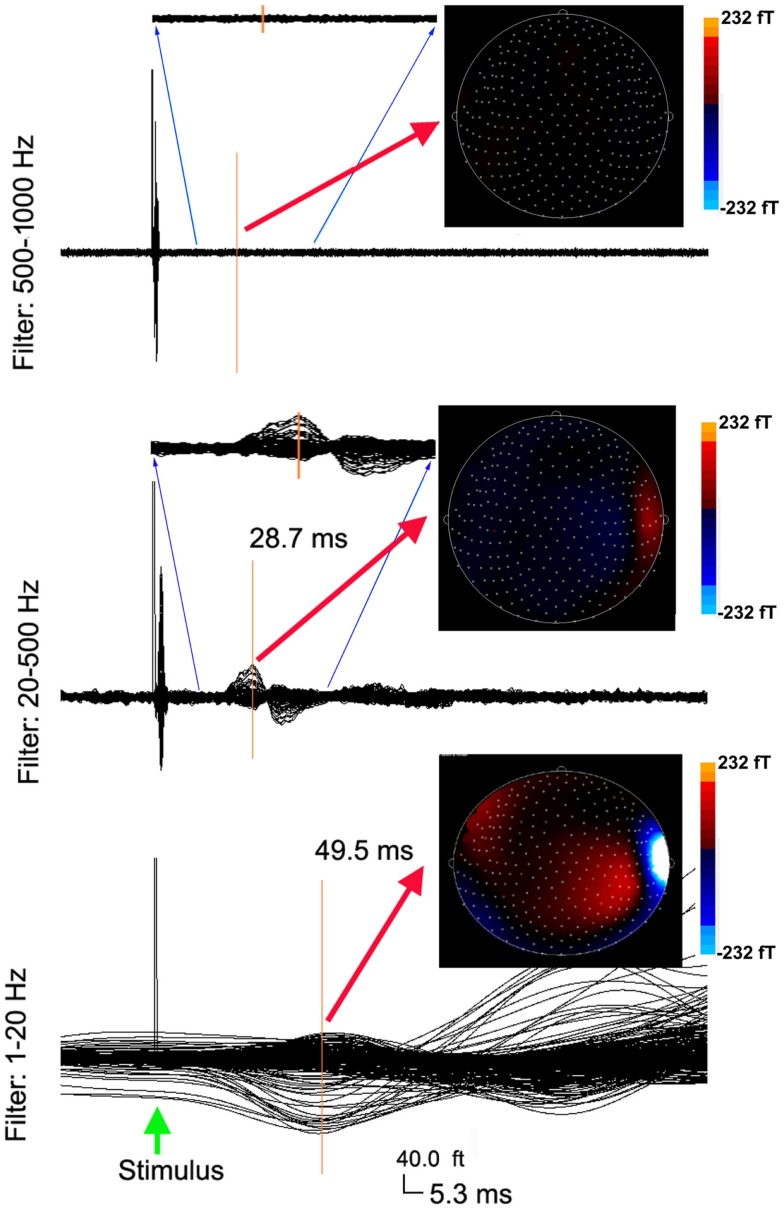
**Averaged waveforms and contour maps from a healthy subject showing neuromagnetic activation in 1–1000 Hz evoked by left finger stimulation**. The three sets of waveforms are filtered by three band-pass filters of 1–20, 20–500, and 500–1000 Hz, respectively. The amplitude of neuromagnetic activation decreases with the increase of frequency range. The latency of neuromagnetic activation shortens with the increase of frequency range. The three sets of waveforms have the same time (“5.3 ms”) and amplitude (“40.0 ft”) scales (the bottom). Waveforms in the 20–500 Hz range and the 500–1000 Hz range reflect a full time scale, as well as a “zoomed in” time scale from 10 to 30 ms to show the detailed deflections. The color contour maps show the distributions of neuromagnetic activation. All the contour maps have the same color scales.

**Figure 2:**
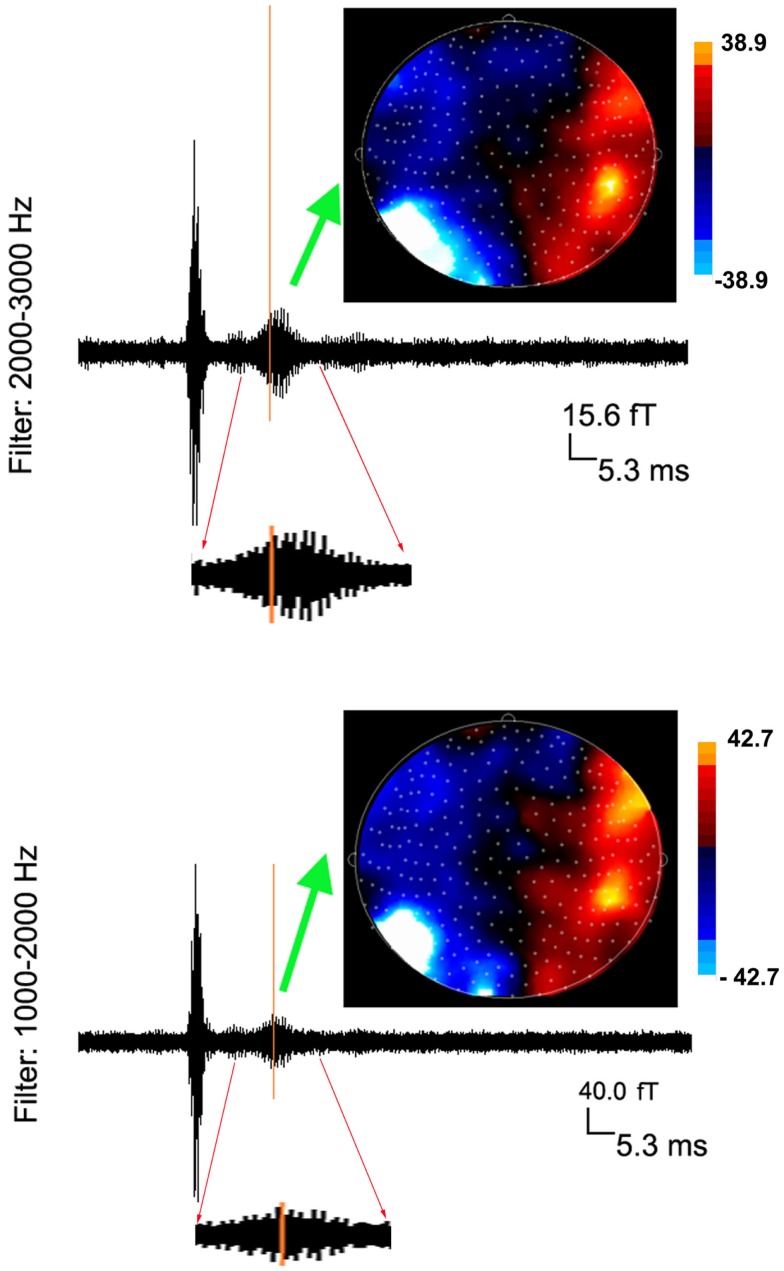
**Averaged waveforms and contour maps from one subject showing neuromagnetic activation in 1000–3000 Hz evoked by left finger stimulation**. The latency of neuromagnetic activation does not change with the increase of frequency range. However, the amplitude of neuromagnetic activation significantly decreases with the increase of frequency range (see the scales). Waveforms from 10 to 30 ms are “zoomed in” to show the detailed deflections (oscillations) elicited by finger stimulation.

**Figure 3:**
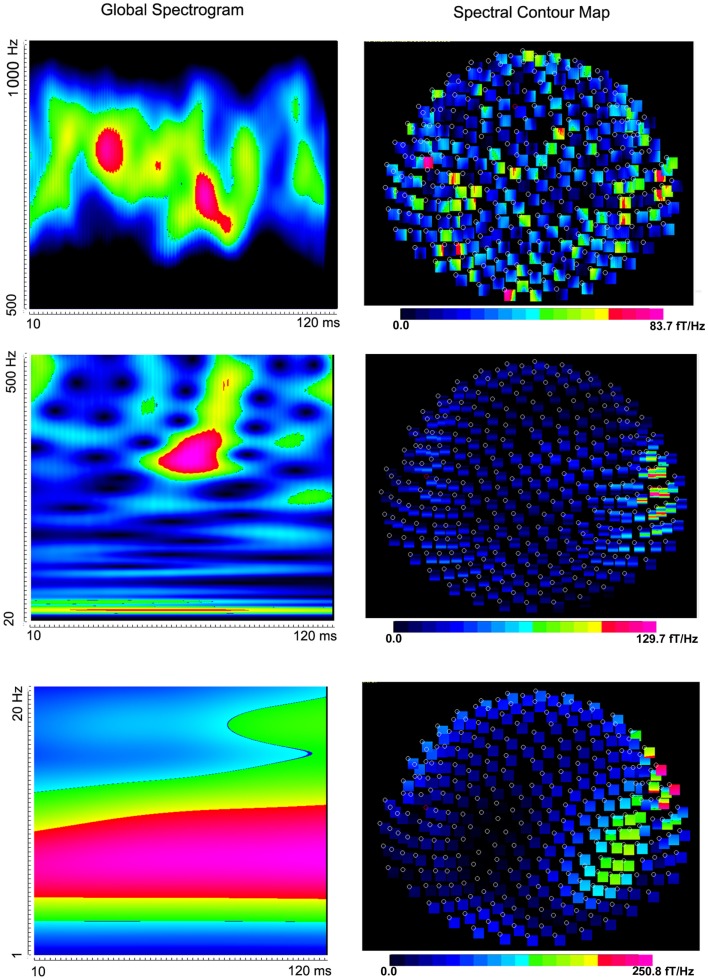
**Global spectrograms and contour maps from one subject showing neuromagnetic activation in 1–1000 Hz elicited by left finger stimulation**. High-frequency spectrograms show precise temporal information while low-frequency spectrograms show precise frequency information.

**Figure 4:**
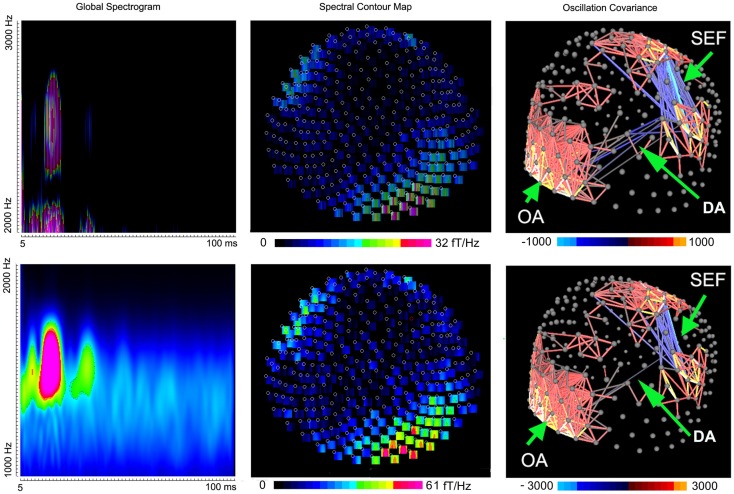
**Global spectrograms, spectral contour maps, and three-dimensional covariance contour maps showing neuromagnetic activation in 1000–3000 Hz elicited by left finger stimulation**. A dipolar source can project signals to sensors to form a dipolar pattern. Mathematically, signals in these “dipolar sensors” are well-correlated and produce a strong oscillation covariance. “SEF” indicates somatosensory evoked magnetic fields; “DA” indicates deep brain activation; “OA” indicates occipital activation.

Somatosensory evoked magnetic fields were analyzed with both band-pass filtering and time-frequency transforms as described above. The amplitude and latency of neuromagnetic responses are summarized in Table 1. We noted that the number of neuromagnetic responses in 500–1000 Hz was less than that in the other four frequency ranges. Figure 1 shows examples of SEFs in the frequency ranges of 1–20, 20–500, and 500–1000 Hz in a healthy subject. Figure 2 shows examples of SEFs in 1000–2000 and 2000–3000 Hz. The amplitude and latency of SEFs in all healthy subjects are shown in Table 1. The result of statistical group analysis revealed that the amplitude of high-frequency signals was significantly weaker (lower) than that of the low-frequency signals (*p* < 0.001). In addition, low-frequency signals appeared in a later latency while high-frequency signals appeared in an earlier latency (*p* < 0.001). Figure 3 shows examples of global spectrograms and spectral contour maps of MEG signals in 1–20, 20–500, and 500–1000 Hz for a healthy subject. Figure 4 shows examples of global spectrograms and spectral contour maps of MEG signals in 1000–2000 and 2000–3000 Hz for a healthy subject. There was no statistically significant difference in amplitude and latency between the left- and right-finger stimulations. We also did not find significant differences in amplitude and latency between the two age groups of subjects (6–11 vs. 12–17 years).

**Table 1:** **Latency and amplitude of somatosensory elicited magnetic fields in multi-frequency ranges (mean ± SD)**.

	Subjects (%)^a^	Latency (ms)	Amplitude (ft)
**Left stimulation**
1–20 Hz	18 (90%)	49.3 ± 3.7	395.6 ± 133.8
20–500 Hz	18 (90%)	28.2 ± 1.8*	168.7 ± 54.3*
500–1000 Hz	9 (45%)	20.5 ± 1.7**	98.3 ± 19.7**
1000–2000 Hz	18 (90%)	19.6 ± 1.4**	52.6 ± 16.2**
2000–3000 Hz	18 (90%)	19.3 ± 1.2**	10.2 ± 6.7**^,^^#^
**Right stimulation**
1–20 Hz	18 (90%)	48.7 ± 3.9	437.5 ± 143.2
20–500 Hz	18 (90%)	27.6 ± 1.7*	179.2 ± 63.6*
500–1000 Hz	9 (45%)	20.8 ± 1.6**	105.3 ± 24.3**
1000–2000 Hz	18 (90%)	20.2 ± 1.4**	62.7 ± 21.5**
2000–3000 Hz	18 (90%)	19.7 ± 1.3**	10.8 ± 7.6**^#^

*^a^Number of subjects that showed a response (deflections)*.

*Compared with 1–20 Hz, *p* < 0.01.

**Compared with 1–20 Hz, *p* < 0.001.

*^#^Compared with 1000–2000 Hz, *p* < 0.01*.

To better understand the spatial distribution, the oscillation covariance was computed for very high-frequency signals in all sensors. The contour map of oscillation covariance showed that very high-frequency signals among sensors over the somatosensory cortex were well-correlated (0.71 ± 0.12, *M* ± SD), which indicated that the signals at the sensors around the somatosensory region were from the same source. Figure 4 shows the examples of three-dimensional contour maps of the oscillation covariance. We also noted that very high-frequency signals in the occipital and frontal sensors were also partially correlated (0.61 ± 0.14, *M* ± SD), which implied deep brain activation (DA). The correlation between the occipital and frontal regions was relatively weak as compared with that in the somatosensory regions (Figure 4, “DA” and “SEF”). Since the spectral power of vHFOs in the occipital and frontal regions was very high, but the sensors in these regions showed a weak correlation (0.13 ± 0.02), those very high-frequency signals were considered to be noise or artifacts. In sum, the data indicated that very high-frequency signals were from the somatosensory cortex and deep brain regions, and may have included some occipital artifacts.

**Figure 5:**
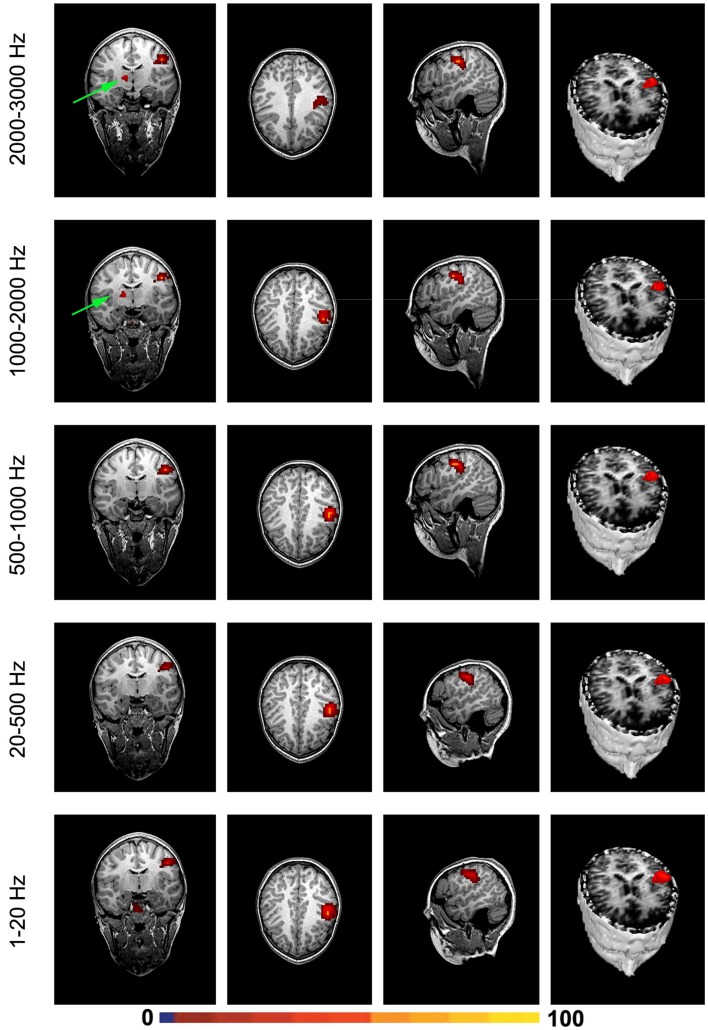
**Magnetic source images showing the sources of brain activation elicited by finger stimulation in a representative subject**. Note that, the somatosensory cortex generates signals in a wide frequency range. Very high-frequency signals (1000–2000 and 2000–3000 Hz) may be also generated by the deep brain area, which is possibly from the thalamus.

**Figure 6:**
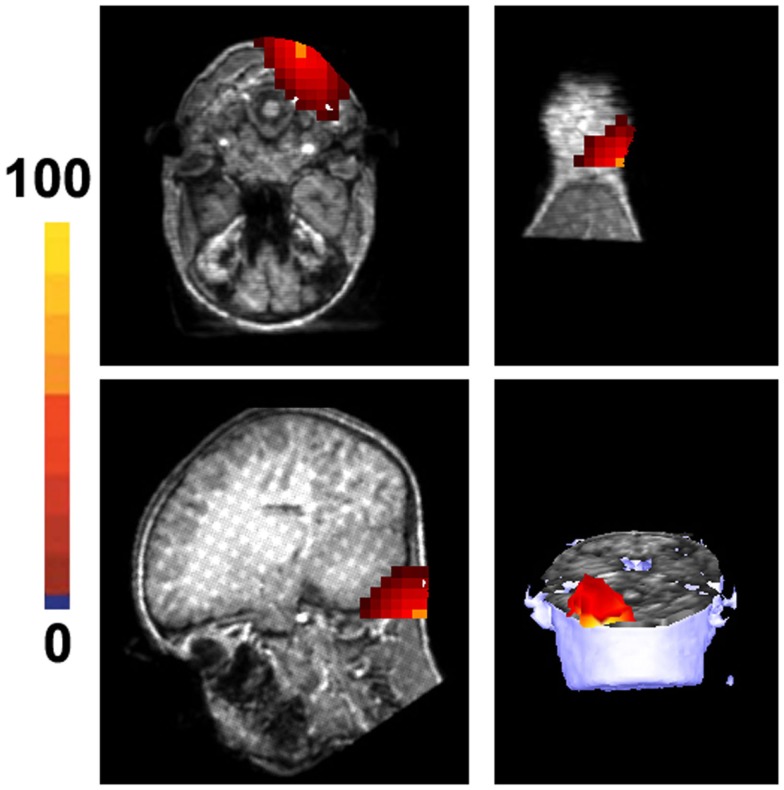
**Magnetic source images show the location of noise in 1000–2000 Hz in a healthy subject**. The noise is from the occipital region and is considered to result from muscle artifacts. Of note, the location of the strongest noise is aligned with the neck and is outside of the brain. The color bar indicates the strength of muscle activity, which is a statistical value that does not have a specific unit.

Magnetic source imaging revealed MEG signals at 1–20 and 20–500 Hz from the somatosensory cortex in 17 subjects (17/20, 85%), MEG signals at 500–1000 Hz from the somatosensory cortex in 6 subjects (6/20, 30%), and MEG signals at 1000–2000 and 2000–3000 Hz from the somatosensory in 18 subjects (18/20, 90%). MEG signals within the range of 1000–2000 and 2000–3000 Hz were also localized to the deep brain area, likely the thalamus, in 18 subjects (18/20, 90%) and 14 subjects (14/20, 70%), respectively. The result of statistical group analysis revealed that the source strength of MEG signals at 1–20 Hz was significantly stronger than that of MEG signals at 2000–3000 Hz in the primary somatosensory cortex (SI) for both left and right stimulation (*p* < 0.001). The result of statistical group analysis did not reveal significantly different SI source coordinates for each of the frequency bands (1–20, 20–500, 500–1000, 1000–2000, and 2000–3000 Hz). However, the source coordinates of the DA in 1000–2000 Hz was significantly different from that of the SI activation (*p* < 0.001). Figure 5 shows the source locations of MEG signals in all the frequency ranges for a healthy subject. The sources of very high-frequency signals around the occipital regions were localized to the posterior regions, which were outside of the brain and were determined to be muscle artifacts. Figure 6 shows an example of one of these muscle artifacts in the posterior regions.

## Discussion

The present study has demonstrated that the developing brain generates high-frequency neuromagnetic signals. Though the cerebral mechanisms underlying neuromagnetic high-frequency signals remain unclear, we noted that stimulation-induced high-frequency neuromagnetic signals were much weaker (lower amplitude) as compared with corresponding low-frequency neuromagnetic signals. Previous literature shows that magnetic signals detected by MEG are typically from ~10,000 to 50,000 synchronously active neurons (Murakami and Okada, 2006). If we assume that the 10,000–50,000 synchronously active neurons comprise a “dipolar source” that can generate a signal strong enough to be recorded by MEG, then high-frequency neuromagnetic signals at the sensor level may be from multiple “dipolar sources.” In other words, the frequency signature of neuromagnetic signals is not equal to the frequency signature of a single neuron firing; instead, the frequency signature of neuromagnetic signals may reflect the spatiotemporal and spectral patterns of multiple sources.

The non-invasive detection of high-frequency brain signals is still a new area of investigation (Gotman, 2010), the reliability and accuracy of the new methods are of utmost concern. We have been careful to eliminate artifacts in the present data. To eliminate possible artifacts, we routinely conducted noise tests just before each clinical and research MEG recording. In addition, the patient’s head position was monitored with three coils and our method volumetrically scanned the sources of the entire brain. In particular, our method was able to localize muscle artifacts to the occipital region. If the high-frequency signals obtained in the present study were artifacts, they should also be localized to a random place or out of the brain. Furthermore, we confirmed these results with time-frequency data, oscillation covariance maps, and MSI. Therefore, it is unlikely that the results reported here are due to the measurement of artifacts. Building on the data from multiple approaches, we conclude that the measurements we have obtained are true neuromagnetic signals in high-frequency ranges.

The finding of high-frequency signals in the somatosensory cortex in the developing brain is consistent with previous reports of HFOs found in the somatosensory cortex in adults (Urasaki et al., 2002; Waterstraat et al., 2012). Compared with previous reports, there are several unique features in the present study. First, previous reports on somatosensory evoked fields were typically recorded by stimulating left and right median nerves (Hashimoto et al., 1999). According to our clinical practice, the finding of median nerves for children can be challenging due to time constraints. Thus, the present study used finger stimulation with 100 trials; a location and trial number more suitable for children. Second, the present study used both the conventional band-pass filters and the newer time-frequency analyses. We demonstrated that the latency and amplitude of neuromagnetic responses to finger stimulation changed with different filters. Third, to our knowledge, this is the first study showing very high-frequency evoked signals (>1000 Hz) from the somatosensory cortex in the developing brain using MEG. Though the present study showed that high-frequency signals could be identified in the somatosensory cortex in children and adolescents, there were no statistical differences between two age groups of subjects (6–11 vs. 12–17 years). Thus, we assume that the developmental changes that occur between these two age groups are minor and/or the number of subjects was not large enough for the difference to reach significance. However, the present results suggest that future studies with a large number of subjects can employ high-frequency MEG signals to study the development of brain function.

The precise frequency ranges of high-frequency neuromagnetic signals from the somatosensory cortex vary among subjects. The cerebral mechanisms of individual variations among subjects in terms of frequency ranges are not yet well understood. Gobbele et al. (2000) have found 600-Hz bursts in the SEFs. We also identified activation in 20–500 and 500–1000 Hz. The percentage of high-frequency bursts in 500–1000 Hz appeared to be lower than that of the other frequency ranges. Though the exact cerebral mechanism remains unclear, we postulate that the stage of brain development and the methodologies used (e.g., stimulation paradigms, data analysis methods) play a role in these findings. Since our results have demonstrated that the developing brain generates high-frequency somatosensory evoked signals, it would be very interesting to standardize MEG protocols for future high-frequency brain signal investigations in children, adolescents, and adults in the future.

Though the main activation was seen in the contralateral somatosensory area (SII) following finger stimulation, we noted deep source activation coming from the ipsilateral thalamus in healthy subjects (see Figure 5, for example). Since the somatosensory tracts are already decussated, the activation in the ipsilateral thalamus might be related to the interhemispherical interactivation of the somatosensory system. One possibility is interhemispherical inhibition. Building on previous reports that finger stimulation is associated with deactivation of the ipsilateral SI (Hlushchuk and Hari, 2006), we postulate that the activation in the ipsilateral thalamus in our data might be related to the ipsilateral SI deactivation. In addition, we consider that activation in the ipsilateral thalamus might be also involved in mediating the activation of the secondary SII ipsilateral to the side of stimulation (Stancak et al., 2002).

As pointed out by Benar et al. (2010), high-pass filtering of waveforms for detection of oscillatory activity should be performed with great care. We have therefore used both filters and time-frequency analyses to verify the HFO findings in the developing brain. Our results from the two approaches strongly suggest that HFOs in the somatosensory system are non-invasively detectable. Of note, MEG HFOs can potentially be viewed as new biomarkers of brain activity, and MEG detection of high-frequency signals may open a new avenue for the study of the brain.

One potential flaw of the present study is the number of stimuli (100). This number was much less that used in previous studies [typically 3,000–5000 stimuli, e.g., Ozaki et al. (1998)]. This shorter paradigm was selected due to the fact that pediatric populations tend to have shorter attention spans than the adults in the previous studies. However, the benefit of the new method (Morelet wavelet, source localization, and accumulated spectrogram) is that it is assumed to require much fewer trials in order to detect high-frequency signals. We recognize the lower trial number as a possible limitation of the present exploratory study. The software and supplementary materials, which implemented the aforementioned algorithms, are freely available from the following website (https://sites.google.com/site/braincloudx/home) for other researchers to test, reproduce, and improve upon.

In summary, the results have demonstrated that somatosensory high-frequency activation can be non-invasively detected with MEG and advanced signal processing methodology. The proposed method was further validated with previously established conventional methods. MEG detection of high-frequency brain activity may open a new avenue in the study of the human brain function in the future.

## Conflict of Interest Statement

The authors declare that the research was conducted in the absence of any commercial or financial relationships that could be construed as a potential conflict of interest.
